# Risk factor analysis of pulmonary cement embolism during percutaneous vertebroplasty or kyphoplasty for osteoporotic vertebral compression fractures

**DOI:** 10.1186/s13018-021-02472-9

**Published:** 2021-05-13

**Authors:** Dexin Zou, Shengjie Dong, Wei Du, Bing Sun, Xifa Wu

**Affiliations:** 1 Department of Spine Surgery, Yantaishan Hospital, Yantai, 264003 Shandong China; 2 Department of Spine Surgery, Zibo Central Hospital, Zibo, No. 54 Gongqingtuan Road, Zhangdian District, Zibo City, Shandong Province, Zibo, 255036 Shandong China

**Keywords:** Vertebroplasty, Kyphoplasty, Pulmonary cement embolism, Osteoporosis, Vertebral compression fracture

## Abstract

**Objective:**

The purpose of this research is to evaluate the risk factors and incidence of pulmonary cement embolism (PCE) during percutaneous vertebroplasty (PVP) or kyphoplasty (PKP) for osteoporotic vertebral compression fractures (OVCFs) based on postoperative computed tomography (CT).

**Methods:**

A total of 2344 patients who underwent PVP or PKP due to OVCFs in our spine center were analyzed retrospectively. According to the detection of postoperative pulmonary CT, the patients were divided into two groups: pulmonary cement embolism group (PCE group) and non-pulmonary cement embolism group (NPCE group). Demographic data in both groups were compared using the *χ*^2^ test for qualitative data and the unpaired *t* test for quantitative data. Multiple logistic regression analysis was carried out to identify risk factors that were significantly related to the PCE resulting from cement leakage.

**Results:**

PCE was found in 34 patients (1.9% 34/1782) with pulmonary CT examination after operation. There was no statistically significant difference in the parameters such as age, gender, body mass index (BMI), and cement volume in the two groups. Patients with three or more involved vertebrae had a significantly increased risk to suffer from PCE than those with one involved vertebra (*p*=0.046 OR 2.412 [95% CI 1.017–5.722]). Patients who suffered thoracic fracture had a significantly increased risk to suffer from PCE than those who suffered thoracolumbar fracture (*p*=0.001 OR 0.241 [95% CI 0.105–0.550]). And significantly increased PCE risk also was observed in thoracic fracture compared with lumbar fracture patients (*p*=0.028 OR 0.094 [95% CI 0.114–0.779]). The risk of PCE within 2 weeks after fracture was significantly higher than that after 2 weeks of fracture (*p*=0.000 OR 0.178 [95% CI 0.074–0.429]). Patients who underwent PVP surgery had a significantly increased PCE risk than those who underwent PKP surgery (*p*=0.001 OR 0.187 [95% CI 0.069–0.509]).

**Conclusion:**

The real incidence of PCE is underestimated due to the lack of routine postoperative pulmonary imaging examination. The number of involved vertebrae, fracture location, operation timing, and operation methods are independent risk factors for PCE.

## Introduction

At present, percutaneous vertebroplasty (PVP) and kyphoplasty (PKP) are widely used in osteoporotic vertebral compression fractures (OVCFs), spinal metastasis, and multiple myeloma [1–3]. This minimally invasive surgery can effectively relieve pain, maintain the stability of the spine, and prevent further collapse and kyphosis of the spine [4–6].

However, it is not absolutely safe; complications related to the operation are often encountered, among which intraoperative cement leakage is the most common complication [2, 3, 7, 8]. Up to now, there are many classification standards about cement leakage, but there is no unified standard at present. The commonly used classification standard is based on the anatomical location and leakage route. The cement can leak into the intervertebral space and paravertebral tissues through fracture fissures, or into the vertebral canal through the basivertebral vein, or into the paravertebral venous plexus through the intravertebral venous network. Though in the majority of cases cement leakage does not cause any clinical syndromes, some severe complications can still be encountered during PVP or PKP procedure, such as neurological deficits and pulmonary cement embolism (PCE) [2, 9–11].

When the injected cement leaks into the paravertebral venous plexus, the cement maybe migrate via segmental spinal veins, inferior vena cava, or azygos vein, eventually passes through the right atrium and ventricle into the pulmonary artery, which can eventually lead to PCE [1, 10]. In the previous literature, most PCE cases during PVP or PKP were reported in the form of case report [12–14]. In fact, most patients with PCE have no clinical symptoms [1, 15], but once there were dyspnea, chest pain, and hemodynamic instability, often indicating poor prognosis or requiring pulmonary thrombectomy or emergency cardiopulmonary resuscitation [13]. Though PCE is a rare complication, many scholars [1, 12, 16, 17] believe that the actual incidence of PCE has been ignored and underestimated due to lack of routine pulmonary image screening after operation. Which factors are closely related to the occurrence of PCE has not been determined [16, 18, 19]. The purpose of this research is to evaluate the risk factors and incidence of PCE during PKP or PVP for OVCFs based on postoperative computed tomography (CT).

## Materials and methods

### Ethics statement

Written informed consent was obtained from each patient, and this study was approved by the Ethics Committee of Yantaishan Hospital and Zibo Central Hospital.

### Patients

From June 2012 to December 2018, a total of 2344 patients who underwent PVP or PKP due to OVCFs in the spine center of Yantaishan Hospital and Zibo Central Hospital were analyzed retrospectively. According to the detection of postoperative pulmonary CT, the patients were divided into two groups: pulmonary cement embolism group (PCE group) and non-pulmonary cement embolism group (NPCE group). The demographic data of the two groups, including age, gender, bone mineral density (BMD), involved vertebrae, and fracture location, are shown in Table 1. Bone mineral density (BMD) was measured by dual energy X-ray absorptiometry (DXA), and osteoporosis was defined by a T-score of ≤ −2.5 SD. Patients with non-osteoporotic vertebral compression fractures, spinal metastases, multiple myeloma, hemangiomas who underwent PKP or PVP surgery, or who were unable to complete pulmonary CT examination after surgery were excluded from this study.

**Table 1:** Demographic data

Item	NPCE	PCE	*t*/*χ*^2^	*p*-value
Case number	1748	34		
Age (years)	71.51±7.11	71.73±7.60	0.1782	0.859
Gender			0.0545	0.815
Male	703	13		
Female	1045	21		
BMD (T-score)	−3.24±0.51	−3.25±0.54	0.1096	0.912
Cement volume (ml)	6.31±0.56	6.56±0.82	2.6401	0.068
Involved vertebrae			9.5355	0.008
One level	936	13		
Two levels	569	10		
Three levels and more	243	11		
Fracture location			21.00	0.000
Thoracic (T5–T9)	427	20		
Thoracolumbar (T10–L2)	1075	13		
Lumbar (L3–L5)	246	1		
Operation timing			16.3292	0.000
Fracture time ≤ 2 weeks	732	26		
Fracture time > 2 weeks	1016	8		
Operation method			15.3862	0.000
PVP	846	28		
PKP	902	6		

*NPCE* non-pulmonary cement embolism, *BMD* bone mineral density, *PVP* percutaneous vertebroplasty, *PKP* percutaneous kyphoplasty

In general, if the patient has no discomfort symptoms after operation, the pulmonary CT examination is generally performed 1 month after operation because the patient will be recommended to return to the hospital for postoperative reexamination and intravenous zoledronic acid for anti-osteoporosis treatment, but if the patient has dyspnea, chest pain, or obvious paravertebral venous cement leakage during or after operation, pulmonary CT examination will be performed immediately. Polymethylmethacrylate (PMMA, Tecres S.P.A., Verona, Italy) was injected into the fractured vertebral body as bone cement in all patients throughout the study.

### Surgical procedures

The PVP or PKP procedure was performed under local anesthesia with the patient in a prone position as previously described. The fractured vertebra was localized in advance under fluoroscopic guidance. In PVP procedure, a disposable 13-gauge needle was introduced from the right side with the unilateral transpedicular approach. After the working cannula was in the appropriate position of fractured vertebra, the drill was advanced to create a tunnel, then removed the drill and injected the PMMA into the vertebra. In contrast, PKP had one more step than PVP. After the drill was removed, an expandable balloon tamp was inserted into vertebral body and then inflated slowly to create a cavity. When the balloon is expanded to achieve the desired effect, the balloon was deflated and removed. The PMMA was injected into fractured vertebra. Meanwhile, the assistant began to prepare the PMMA at the room temperature, divided them into several 1-ml syringes, and then put them into iced water to prolong the PMMA coagulation time. When the PMMA consistency looked mild toothpaste-like, then started to inject. At the beginning of injection, only 0.1 ml PMMA was injected into the vertebra, then checked the position of PMMA under fluoroscopy to exclude the leakage. The injection volume was increased to 0.2 ml and repeated the same procedure. Then, each injection volume was increased to 0.3 ml and was not added any more. If there was a small amount of paravertebral PMMA leakage during the operation, generally stop the operation for 10–15 s, and then resume the PMMA injection. If there was a large amount of PMMA leakage, stop the operation immediately, no matter where the operation goes.

### Statistical analysis

Statistical analysis was performed with SPSS 20.0 (SPSS In., Chicago, IL, USA). All measurement data were described as mean and standard deviation (SD). Demographic data in both groups were compared using the *χ*^2^ test for qualitative data and the unpaired *t* test for quantitative data. Multiple logistic regression analysis was carried out to identify risk factors that were significantly related to the PCE resulting from cement leakage. The *P*-value < 0.05 was considered statistically significant.

## Results

### Baseline characteristics of the patients

Among a total of 2344 patients, 1782 patients met the inclusion criteria. The NPCE group consisted of 1748 patients (703 males and 1045 females), with ages ranging from 56 to 88 years (mean, 71.51±7.11 years), BMD with T-value ranging from −2.5 to −4.6 SD (mean, −3.24±0.51 SD), and injected cement volume of single vertebral body ranging from 4.2 to 8.5 ml (mean, 6.31±0.56 ml). By contrast, the PCE group consisted of 34 patients (13 males and 21 females), with ages ranging from 57 to 85 years (mean, 71.73±7.60 years), BMD with T-value ranging from −2.6 to −4.9 SD (mean, −3.25±0.54 SD), and injected cement volume of single vertebral body ranging from 4.0 to 8.7 ml (mean, 6.56±0.82 ml). There was no statistically significant difference in the parameters mentioned above in the two groups (Table 1).

**Fig. 1:**
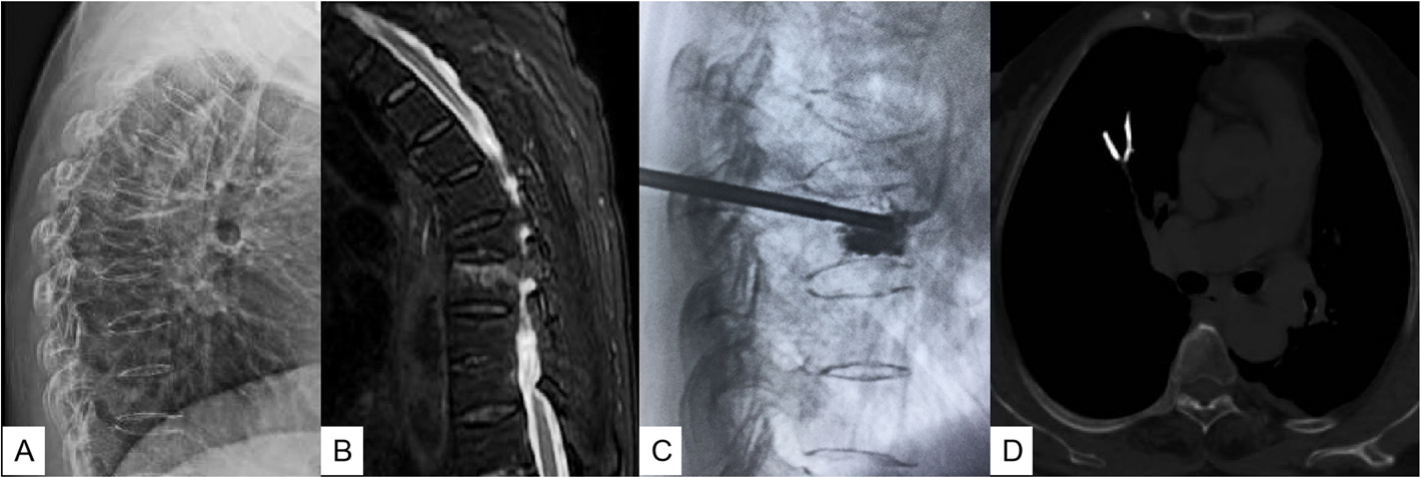
A 72-year-old woman was hospitalized complaining of severe back pain after falling down 2 weeks before admission. Preoperative thoracic lateral X-ray showed T7 vertebral compression fracture (**a**). Sagittal MRI (fat-suppressed T2-weighted images) showed that T7 was old fracture, while T8 was new osteoporotic compression fracture (**b**). Intraoperative thoracic lateral fluoroscopy showed that bone cement spilled into the anterior paravertebral vessels (**c**). Postoperative pulmonary CT found the pulmonary cement emboli of right inferior lung lobe (**d**)

PCE was found in 34 patients (1.9% 34/1782) with pulmonary CT examination after operation. Among them, 6 patients had transient dyspnea, chest pain, or hemodynamic instability during the operation, and the symptoms were relieved after supportive treatment and anticoagulation. The remaining 28 patients did not have any discomfort and were not given any treatment. They were followed up regularly. No critical patients need surgical treatment or cause death. In all the 34 patients, pulmonary emboli were located in the subsegmental or peripheral arteries, and no central pulmonary artery embolism was found (Fig. 1).

### Analysis of the risk factors between the PCE group and NPCE group

In the location of vertebral fracture, the incidence of PCE in thoracic spine was 4.9% (20/427), in thoracolumbar spine was 1.2% (13/1075), and in lumbar spine was 0.4% (1/246). In the choice of operation timing, the incidence of PCE with fracture time less than 2 weeks was 3.6% (26/732), and that of more than 2 weeks was 0.8% (8/1016). In the selection of operation method, in PKP group, the incidence of PCE was 3.3% (28/848), and PVP was 0.7% (6/902). The abovementioned data differences were statistically significant (Table 1).

### The logistic regression analysis of the risk factors

The parameters included in the multiple logistic regression model were involved vertebrae, fracture location, operation timing, and operation method. Patients with three or more involved vertebrae had a significantly increased risk to suffer from PCE than those with one involved vertebra (*p*=0.046 OR 2.412 [95% CI 1.017–5.722]). But there was no significant increase in the risk of PCE in two involved vertebrae compared with one involved vertebra (*p*=0.56 OR 1.278 [95% CI 0.560–2.918]). Patients who suffered thoracic fracture had a significantly increased risk to suffer from PCE than those who suffered thoracolumbar fracture (*p*=0.001 OR 0.241 [95% CI 0.105–0.550]). And significantly increased PCE risk also was observed in thoracic fracture compared with lumbar fracture patients (*p*=0.028 OR 0.094 [95% CI 0.114–0.779]). In the timing of operation, the risk of PCE within 2 weeks after fracture was significantly higher than that after 2 weeks of fracture (*p*=0.000 OR 0.178 [95% CI 0.074–0.429]). Patients who underwent PVP surgery had a significantly increased PCE risk than those who underwent PKP surgery (*p*=0.001 OR 0.187 [95% CI 0.069–0.509]) (Table 2).

**Table 2:** Risk factors for PCE using multiple logistic regression analysis

Variable	OR	ST	*Z*	*P*>∣Z∣	95% CI
Involved vertebrae					
1	1.278	0.538	0.58	0.560	0.560–2.918
2	2.412	1.063	2.000	0.046	1.017–5.722
Fracture location					
1	0.241	0.101	−3.38	0.001	0.105–0.550
2	0.094	0.102	−2.19	0.028	0.114–0.779
Operative timing	0.178	0.798	−3.85	0.000	0.074–0.429
Operation method	0.187	0.096	−3.29	0.001	0.069–0.509

*PCE* pulmonary cement embolism, *OR* indicates odds ratio, *ST* stand error, *CI* confidence interval

## Discussion

Nowadays, with the aggravation of population aging, osteoporotic vertebral compression fractures (OVCFs) have gradually become a worldwide public health problem [8, 20]. Conservative management, such as analgesics, bed rest, and orthoses, used to be the main treatment method of OVCFs [21], but with the clinical application of cementoplasty techniques, treatment options are gradually changing. At present, although there is still controversy regarding the superiority of cementoplasty techniques over conservative management [20, 21], PVP or PKP can provide immediate pain relief, reconstruction of spinal stability, and significantly improve quality of life, so these become more and more popular in the treatment of patients with OVCFs [1–3]. Though they are minimally invasive procedure, complications still exist. According to the literature [2, 3, 7, 8], cement leakage is the most common complication. Once the PMMA leaks into the paravertebral venous plexus, the PMMA maybe migrate via segmental spinal veins, inferior vena cava, or azygos vein, eventually to pulmonary artery and induce the PCE [1, 10, 22–24].

PCE is a rare complication; most PCE cases during PVP or PKP procedure were reported in the form of case report [12–14]. In the previous literature, the incidence of PCE ranged from 0.3 to 23% [14, 18, 24]. However, many scholars [1, 12, 14] still believe that the actual incidence of PCE has been ignored and underestimated due to lack of routine pulmonary imaging screening after operation. In our study, the incidence of PCE was 1.9% based on the postoperative pulmonary CT scan. We believe that in addition to postoperative imaging screening, there are other risk factors closely related to the occurrence of PCE.

There was no significant difference in age, gender, and BMD between PCE group and NPCE group. Although theoretically speaking, the more bone cement is injected, the higher the probability of PCE will appear, but according to our statistics, the total bone cement filling amount of PCE group is not significantly increased than that of NPCE group (*p*=0.068).

From the data of logistic regression analysis, when two vertebral segments were involved, compared with one segment, there was no significantly statistical difference in the occurrence of PCE (*p*=0.560), but when three or more segments were involved, the significantly statistical difference was observed compared with one segment (*p*=0.046). The more vertebral segments involved, the greater the probability of PCE. First of all, more involved segments suggest that more bone cement should be injected during the PVP or PKP procedure, and the probability of leakage is significantly increased. Secondly, long-time prone position leads to the decrease of cardiopulmonary compensatory function in elderly patients with osteoporosis, which can easily cause hemodynamic instability and increase the risk of surgery. Therefore, it is not recommended that more than three vertebral segments were filled with bone cement at a time.

In terms of fracture location, thoracic vertebrae occupied 20 patients, thoracolumbar spine accounted for 13 patients, lumbar spine occupied only 1 patient, and the risk of PCE gradually decreased from thoracic spine to lumbar spine. This is mainly related to the anatomical characteristics of the spine. First of all, compared with lumbar vertebrae, thoracic vertebrae are smaller, and pedicle is thinner, so surgical puncture is difficult, and bone cement leakage is more common. Secondly, thoracic vertebrae are close to cardiopulmonary vessels. Once paravertebral cement leakage occurs, unpolymerized bone cement can quickly enter the heart, and then reach the pulmonary artery to cause PCE, while lumbar bone cement leakage needs to go through longer paravertebral vein or lumbar ascending vein to the inferior vena cava or azygos vein; sometimes bone cement has polymerized in the vertebral venous system before reaching the larger vein.

Our statistical results confirm that the operation timing of OVCFs is an independent risk factor for PCE. Although some studies have shown that early surgical treatment of OVCFs has advantages in relieving pain symptoms, restoring the height of fractured vertebral body, and reducing the risk of long-term adjacent segment fracture [25], Guan et al. [26] found that both early (≤ 2 weeks) and delayed (> 2 weeks) operations of kyphoplasty can achieve satisfactory outcomes for OVCFs, but the risk of cement leakage during kyphoplasty will decrease obviously in delayed operation. Our study also suggests that if operation is performed more than 2 weeks after fracture, the risk of PCE will be significantly reduced. After osteoporotic fracture, the integrity of basivertebral veins is damaged. If PVP or PKP is performed early, bone cement will easily enter the injured basivertebral veins and its connections, then cause paravertebral vascular embolism or even PCE.

In the PVP group, the incidence of PCE was 3.30% (28/846), while in the PKP group the incidence of PCE was only 0.67% (6/902). As an improved surgical procedure, PKP has been widely mentioned in the previous literature [14, 26–28]. The balloon inflation can not only restore the height of the vertebral body to a certain extent, but also compact the surrounding cancellous bone in the process of balloon expansion, form a cavity in the vertebral body, reduce the perfusion pressure of bone cement, seal the osseous defects, and make the leakage rate of bone cement significantly lower than that of PVP.

In addition to the risk factors mentioned above, the other operation details may also be the potential risk factors of PCE that we cannot ignore, including the viscosity of bone cement, injection pressure, and the amount of cement per single time. Although the thin cement is conducive to the dispersion in the vertebral body, it is also easy to cause the leakage of cement. The leakage probability of viscous bone cement is reduced, but the bone cement cannot be fully dispersed between the bone trabeculae and affect the postoperative clinical effect. Under the same solidification state of the bone cement, higher injection speed and injection pressure are theoretically more likely to cause leakage of the bone cement. However, if the bone cement is more viscous, higher injection pressure will be required to achieve the same injection speed, so there is a close relationship between them. These are also the risk factors that cannot be ignored in clinical practice. At present, it is generally recommended to use cement in toothpaste-like state [17, 23, 29], which can not only give consideration to dispersion, but also have relatively lower leakage of cement. Although in our study there was no significant difference in the total injected amount of cement between the PCE group and the NPCE group, it is suggested that the total amount of cement should be divided into multiple injections under X-ray fluoroscopy during the operation. It is recommended that the single injected volume should not exceed 0.3ml, so that even if there is leakage of cement, patients will not have serious complications. In order to prevent the uninjected cement from coagulating at room temperature, it can be stored in iced water to prolong its coagulation time. Perhaps it is because of these operational details that the incidence of PCE in our study is relatively low, and there is no fatal pulmonary embolism.

At present, there is no standard guideline for the treatment of PCE patients [1, 18, 29]. Optimal treatment depends on the severity of symptoms and the location of the cement embolus. For PCE patients with severe symptoms, most scholars still recommend surgical removal of embolus, followed by anticoagulant therapy [16, 22]. For patients with mild symptoms, anticoagulation and observation are the main method. However, there is no consensus on the specific anticoagulant time. Janssen et al. [17] suggests that patients with mild or asymptomatic PCE were all treated with anticoagulation for at least half a year. In our study, there were 34 patients with PCE, and only 6 patients had discomfort. After supportive treatment, we continued to give oral anticoagulant (rivaroxaban, Bayer Phama AG, Germany) for 1 month. If the patient had no any discomfort, the anticoagulant drugs would be stopped. Other 28 asymptomatic PCE patients were found 1 month after operation when they returned to the hospital for intravenous zoledronic acid to delay the progress of osteoporosis, no further anticoagulants were given due to no any discomfort. Up to now, there are no patients who need to return to the hospital for further treatment due to PCE. The specific anticoagulant time of PCE is still questionable [17, 28].

Our study also has its own limitations. First of all, this is a retrospective study, and there is a certain selective bias. We only retrospectively analyzed the patients who underwent pulmonary CT examination after surgery, and the detection time of postoperative CT varies according to whether the patients have discomfort or not. Secondly, because patients with OVCFs often have calcification of cardiopulmonary vessels, sometimes it is difficult to distinguish them from bone cement embolism images under CT scan, which has a certain impact on our diagnosis. If pulmonary CT scan can be performed before operation, the influence of vascular calcification can be eliminated by preoperative and postoperative comparison. Besides, in addition to the risk factors mentioned above, other risk factors such as the viscosity of bone cement and injection velocity are also factors that cannot be ignored. However, these factors cannot be specifically quantified, so we can only try our best to maintain their consistency during PKP/PVP procedure to minimize the impact on the study. Nevertheless, these factors still had some influence on our study.

## Conclusion

PCE is a rare but serious complication in PKP or PVP. Its real incidence is underestimated due to the lack of postoperative pulmonary imaging examination. The number of involved vertebrae, fracture location, operation timing, and operation methods are independent risk factors for PCE. In order to reduce the incidence of PCE, the above risk factors should be fully considered before operation. Other risk factors associated with PCE and the duration of anticoagulation after PCE are still unclear, so further multicenter prospective studies and long-term follow-up are recommended.

## Data Availability

The datasets used and/or analyzed during the current study are available from the corresponding author on reasonable request.

## Abbreviations

PCE: Pulmonary cement embolism
PVP: Percutaneous vertebroplasty
PKP: Percutaneous kyphoplasty
OVCF: Osteoporotic vertebral compression fracture
CT: Computed tomography
PMMA: Polymethylmethacrylate
BMD: Bone mineral density
DXA: Dual energy X-ray absorptiometry
